# Helping people, neglecting soil: contrasting effects of emotional empathy with humans on altruistic behaviour and soil conservation in a population of Chilean farmers

**DOI:** 10.3389/fpsyg.2026.1843293

**Published:** 2026-07-09

**Authors:** Alexander Neaman, Pamela Pensini, Claudia Navarro-Villarroel, Dmitry S. Ermakov, Mónica Castro

**Affiliations:** 1 Facultad de Ciencias Agronómicas, Universidad de Tarapacá, Arica, Chile; 2 School of Psychological Sciences, Monash University, Melbourne, VIC, Australia; 3 Facultad de Ciencias, Universidad de Valparaíso, Valparaíso, Chile; 4 Department of Psychology and Pedagogy, Peoples’ Friendship University of Russia Named After Patrice Lumumba (RUDN University), Moscow, Russia; 5 Escuela de Agronomía, Pontificia Universidad Católica de Valparaíso, Quillota, Chile

**Keywords:** affective empathy, altruism, environmental psychology, Rasch modelling, soil conservation

## Abstract

The empathy-altruism hypothesis posits empathy is a pre-requisite for altruistic behaviour. Similarly, proposed an empathy-sustainability hypothesis, suggesting empathy is a pre-requisite for sustainable interactions with nature. However, the existing literature tends to intermix the concepts of empathy with humans and empathy with nature. In this study, we aimed to assess the effects of emotional empathy with humans on altruistic behaviour and soil conservation behaviour, in a population of Chilean farmers (*n* = 234). Although the findings of the present study support the empathy-altruism hypothesis (*r* = 0.49), they do not support the empathy-sustainability hypothesis, since a negative correlation emerged between emotional empathy and soil conservation behaviour (*r* = −0.24). This correlation was pronounced in male farmers (*r* = −0.37) and not in female counterparts (*r* = −0.08). The present study suggests that fostering emotional empathy with humans may not be helpful in solving current soil degradation issues.

## Introduction

Empathy is defined as “*understanding a person from their frame of reference rather than one’s own, or vicariously experiencing that person’s feelings, perceptions, and thoughts*” (APA, 2026). In accordance with the empathy-altruism hypothesis (Batson et al., 1991), empathy is considered a prerequisite for altruistic behaviour (Batson et al., 2015; Cialdini et al., 1997). The effect of empathy in motivating prosocial behaviours is attributed to self-other overlap, which is an expansion of self that incorporates other people (Brethel-Haurwitz et al., 2018; Dong et al., 2025; Feng et al., 2020).

Similar to the empathy-altruism hypothesis, Brown et al. (2019) proposed an empathy-sustainability hypothesis, suggesting that empathy is required for sustainable interactions with nature because it provides a motivation for pro-environmental behaviours. Notably, Brown et al. (2019) proposed combining the analysis of empathy with humans and empathy with the natural world, termed “ecological empathy” (Lambert, 2024) or “dispositional empathy with nature” (Tam, 2013) or “eco-empathy” (El Refaie and Thatcher, 2025) or “empathy with nature” (Raymond et al., 2025), hereafter “empathy with nature.” Recent work has examined empathy with nature in relation to tourists’ responsible behavioural intentions in natural heritage tourism (Wang and Lu, 2025; Wang et al., 2025), pro-environmental behavioural intention in digital-media contexts (Chen et al., 2024), and conservation behaviour in public and private spheres (Raymond et al., 2025).

However, the following question arises: Is empathy with humans a prerequisite of pro-environmental behaviours? There is no clear answer to this question because the existing literature tends to intermix the concepts of empathy with humans and empathy with nature, as exemplified in the review of Luo et al. (2025). For instance, the study of Berenguer (2010) demonstrates that empathy is related to the number of moral reasons given for pro-environmental behaviours. However, this study also intermixes empathy with humans and empathy with nature, making it difficult to understand the underlying mechanisms.

It should be noted that empathy with nature is not necessarily linked with empathy with humans (Raymond et al., 2025), suggesting that empathy toward humans should be treated as conceptually distinct from empathy with nature, which is not the case in the current literature.

Another complexity with the concept of “empathy with humans” is related to the distinction between emotional empathy (feeling others’ emotions) and cognitive empathy (understanding others’ emotions) (Perry and Shamay-Tsoory, 2013). For brevity, in the following discussion, the terms “emotional empathy” and “cognitive empathy” will refer exclusively to empathy with humans. Although some studies used the term “affective empathy” (e.g., Ienna et al., 2022), hereafter we will use the term “emotional empathy” because it is more common in the literature.

The study of Ienna et al. (2022), on a sample of general population in Australia, demonstrated that environmental preservation behaviours were positively associated with cognitive empathy, but not emotional empathy. Likewise, the study of Raymond et al. (2025), on a sample of general population in Finland, demonstrated that cognitive empathy was a positive predictor of household conservation actions, whereas the interaction term cognitive empathy × emotional empathy was a negative predictor of such actions. In contrast, Neaman et al. (2022) demonstrated positive correlation between emotional empathy and pro-environmental behaviour in a sample of general population in Latin America (Chile, Mexico, and Guatemala) and a sample of general population in Russia.

These contradicting results can be due to the fact that emotional empathy is known to predict altruistic behaviour (Bethlehem et al., 2017), which in turn is closely related to pro-environmental behaviour (Neaman, 2025a; Neaman, 2025b). Thus, the effect of emotional empathy on pro-environmental behaviour can be potentially due to shared variance with altruistic behaviour. In contrast, soil conservation behaviour, i.e., specific actions taken by farmers to minimize soil degradation (Jalilian et al., 2025), was not correlated with altruistic behaviour in a population of Chilean farmers (yet unpublished data). Thus, testing the empathy-sustainability hypothesis using farmers’ soil conservation behaviour might offer an advantage due to the expected absence of correlation with altruistic behaviour.

Notably, soil degradation is a global challenge (Editorial, 2004). Addressing this problem requires looking beyond technology and regulations to understand psychological factors that shape behaviour. Yet, despite the vital role of soil for civilization, which is historically linked to societal collapse (Montgomery, 2012) and essential for sustainable development (Bouma et al., 2021), our understanding of why farmers do, or do not, adopt sustainable practices is limited (Winkler-Schor et al., 2024). Therefore, studying the determinants of soil conservation behaviour is an interesting and relevant topic *per se*.

Finally, it should be noted that females are more empathetic than males (Christov-Moore et al., 2014; Jolliffe and Farrington, 2006; Raymond et al., 2025). However, in a prior Chilean study (Neaman et al., 2025), there were no gender differences in soil conservation behaviour. In other words, literature reports differential gender effects on respondents’ emotional empathy and soil conservation behaviour.

### Hypothesis

Based on the aforementioned empathy-altruism and empathy-sustainability hypotheses, we assumed that empathy affects behaviour and not vice versa. We hypothesized that farmers’ emotional empathy will strengthen their engagement in both altruistic and soil conservation actions.

Likewise, we assumed that there might be differential gender effects on respondents’ emotional empathy and engagement in soil conservation, causing a moderation effect of gender on the relationship between emotional empathy and soil conservation actions.

### Objectives

We aimed to assess the relationship of emotional empathy with altruistic behaviour and soil conservation behaviour, in a population of Chilean farmers. We also evaluated how respondents’ gender influences the relationship between emotional empathy and soil conservation actions.

## Methods

### Population

This study involved 234 Chilean farmers from the Los Lagos Region and Los Rios Region (southern Chile), Valparaiso Region (central Chile), and the Arica and Parinacota Region (northern Chile). With 234 total participants, we were able to detect a correlation of *r* = 0.13, with alpha = 0.05 (two tailed) (Faul et al., 2007). For separate correlations by gender, it should be noted that there were 121 male and 111 female participants. With *n* = 111, we were able to detect a correlation of *r* = 0.19, with alpha = 0.05 (two tailed) (Faul et al., 2007).

The term “farmers” in this study included persons who were responsible for making independent decisions related to farm soil management. This included the following groups: independent farmers (73%), agricultural company employees (7%), agricultural company owners (10%), and those who did not specify belonging to any of these three groups (10%), regardless of farm size or their possession (or lack thereof) of a professional degree in agriculture.

While data obtained from convenience sampling cannot be generalized to the larger population, representative sampling accurately reflects the total population (APA, 2026). Therefore, obtaining a representative sample of Chilean farmers would be ideal. However, this task is challenging because of the specific eligibility criteria required for the purpose of this study (persons who were responsible for making independent decisions related to farm soil management). The information for this subgroup of Chilean farmers is missing.

Nevertheless, the Chilean agricultural census (INE, 2022) specifies that there are 130,000 agricultural producers in Chile, of which 68% are males, and of which 53% are of indigenous ethnicity. Likewise, the census reports the prevalence of the following age groups among Chilean farmers (years old): 18–24 – 1%, 25–49 – 22%, 50–64 – 39%, >65–38%. However, the census lacks information on the distribution of independent farmers, agricultural company employees, and agricultural company owners.

Of the total farmers in the present study, 52% are males, and 47% are indigenous, with age ranging between 18–82 years old (*M* ± *SD* of 49 ± 15). Thus, the population under study is quite representative of the Chilean farmers’ population in terms of gender, indigenous ethnicity, and age (Supplementary Table S1).

### Procedure

To recruit this sample in the aforementioned regions, a surveyor toured agricultural areas, stopping at farms. The farmer was invited to complete a paper-and-pencil questionnaire. No incentives were offered to participate. Written informed consent was obtained from all individual adult participants included in the study.

After the farmer completed the questionnaire, the surveyor requested a farm tour, for the purpose of observing practices, asking specific questions, and noting observable behaviours on a validation form, which was then attached to the questionnaire.

### Measures

Data was collected through self-report surveys that included three scales: (1) emotional empathy, (2) soil conservation behaviour, and (3) altruistic behaviour.

Sociodemographic information, including age, gender, indigenous status, whether they grew up in the countryside or urban setting, have formal education in soil management, and membership in humanitarian and environmental organisations was also captured. In addition to describing the sample, this information is utilised to provide additional validity criteria for each scale based on its relationship with these sociodemographic variables.

1) Emotional empathy: We created a Rasch-type scale to quantify emotional empathy. Applying an item-response-theoretical measurement approach allowed us to cover a broad range of item difficulties (Bond and Fox, 2007) which is especially useful for a differentiation between participants with varying levels of the measured concept of interest (e.g., empathy). For this reason, in this study, a Rasch-type model was preferred over a measurement approach according to Classical Test Theory.

Specifically, we chose items from existing empathy scales, namely the Basic Empathy Scale (Jolliffe and Farrington, 2006), a measure of Emotional Empathy for Adolescents and Adults (Caruso and Mayer, 1998), the Perth Empathy Scale (Brett et al., 2023), and Toronto Empathy Questionnaire (Spreng et al., 2009). Item selection criterion was to include items that reflected only emotional empathy (e.g., “When I see or hear someone who is embarrassed, it makes me feel embarrassed too”) and excluded items representing cognitive empathy (e.g., “Just by seeing or hearing someone, I know if they are feeling embarrassed”). Another item selection criterion was to include items of different difficulty level, based on the evaluation of an independent expert. Likewise, translation process involved help of professional Spanish editor, familiar with Chilean culture.

Likewise, we included in our scale the four items of the sentimentality scale of the HEXACO personality inventory (Lee and Ashton, 2018). Higher sentimentality scores relate to greater emotional empathy because sentimentality denotes to empathic sensitivity (Ashton and Lee, 2007). Notably, in our previous study in Spanish (Neaman et al., 2022) emotional empathy was operationalized through this sentimentality scale. The resulting scale is shown in Supplementary Tables S2a,b.

2) Soil conservation behaviour: The Spanish-language scale from Neaman (2024) (Supplementary Tables S3a,b). The validity criteria for this scale obtained in prior Chilean studies are presented in Supplementary Material.

3) Altruism: The altruism scale from Otto et al. (2021) in Spanish (Supplementary Tables S4a,b), adapted from the original self-report altruism scale by Rushton et al. (1981) was used. The validity criteria for this scale obtained in prior Chilean studies are presented in Supplementary Material.

### Data analysis

The Rasch model was used to compute scores of the empathy scale and soil connection scale using the TAM package within RStudio (R Core Team, 2021). The reliability was estimated based on the Rasch model. Supplementary Tables S2a, S3a, and S4a present item difficulties in logits. Larger logit values represent higher scores on each corresponding scale. Likewise, Supplementary Tables S2a, S3a, and S4a present the values of infit mean square (MS), which is a fit statistic used in Rasch modelling to assess whether the responses to a given item conform to model predictions. Values of MS ≤ 1.2 are considered good, while 1.2 < MS ≤ 1.3 are considered acceptable (Wright et al., 1994). To test the unidimensionality of the empathy and soil connection scales, we conducted a principal components analysis of the Rasch residuals (Brentani and Golia, 2007).

Based on participants’ Rasch scores, Pearson’s correlations were computed (Table 1). Likewise, the relationships between empathy and behavioural measures were further examined by controlling for sociodemographic characteristics (age, gender, educational level, income level, indigenous status, and membership in humanitarian and environmental organizations). Gender, indigenous status, and membership in humanitarian and environmental organizations were dummy coded, whereas education and income levels were considered ordinal.

**Table 1:** Pearson correlations between the variables under study.

Variable	1	2	3
1. Empathy	–		
		
2. Soil conservation behaviour	−0.24^***^	–	
	[−0.12, −0.36]		
3. Altruistic behaviour	0.49^***^	n.s.	–
	[0.38, 0.58]		

Values in square brackets indicate the 95% confidence interval for each correlation. n.s., not statistically significant, ^***^ *p* < 0.001.

Furthermore, to examine the effect of sociodemographic characteristics on variables under study, t-test was performed. Individuals who did not specify their gender were excluded from the *t*-tests. It should be noted that for studies with a large sample size (like *n* = 234 in the present study), *t*-tests can be used regardless of whether data distribution is normal or non-normal (Cessie et al., 2020; Fagerland, 2012; Sainani, 2012; Tsagris and Pandis, 2021).

It should be noted that differential item functioning analysis is a statistical technique used to ensure the equity and fairness of assessment (Wyse and Mapuranga, 2009). Differential item functioning can arise across a variety of demographic groups, including gender (Wedman, 2018). It occurs when sample subgroups respond differently to an individual item despite equal levels of ability. In this study, gender-related differential item functioning in the emotional empathy scale was tested using the difR package within RStudio (Magis et al., 2010).

## Results

### Scale reliability, unidimensionality, and validity

The separation reliability estimated based on the Rasch model results was 0.81 for the empathy scale, 0.78 for the soil conservation behaviour scale, and 0.83 for the altruistic behaviour scale. All questions on these scales had good item fit, except for one item of the empathy scale with still acceptable fit (Supplementary Table S2a).

The Rasch residuals explained 9.4, 5.7, and 9.5% of the variance for the scales of empathy, soil conservation behaviour, and altruistic behaviour, respectively. This suggests that the residuals did not contain any significant additional dimensions beyond the core constructs of interest (Fisher, 2007). Therefore, each of our scales effectively measured the single underlying construct of interest.

**Figure 1:**
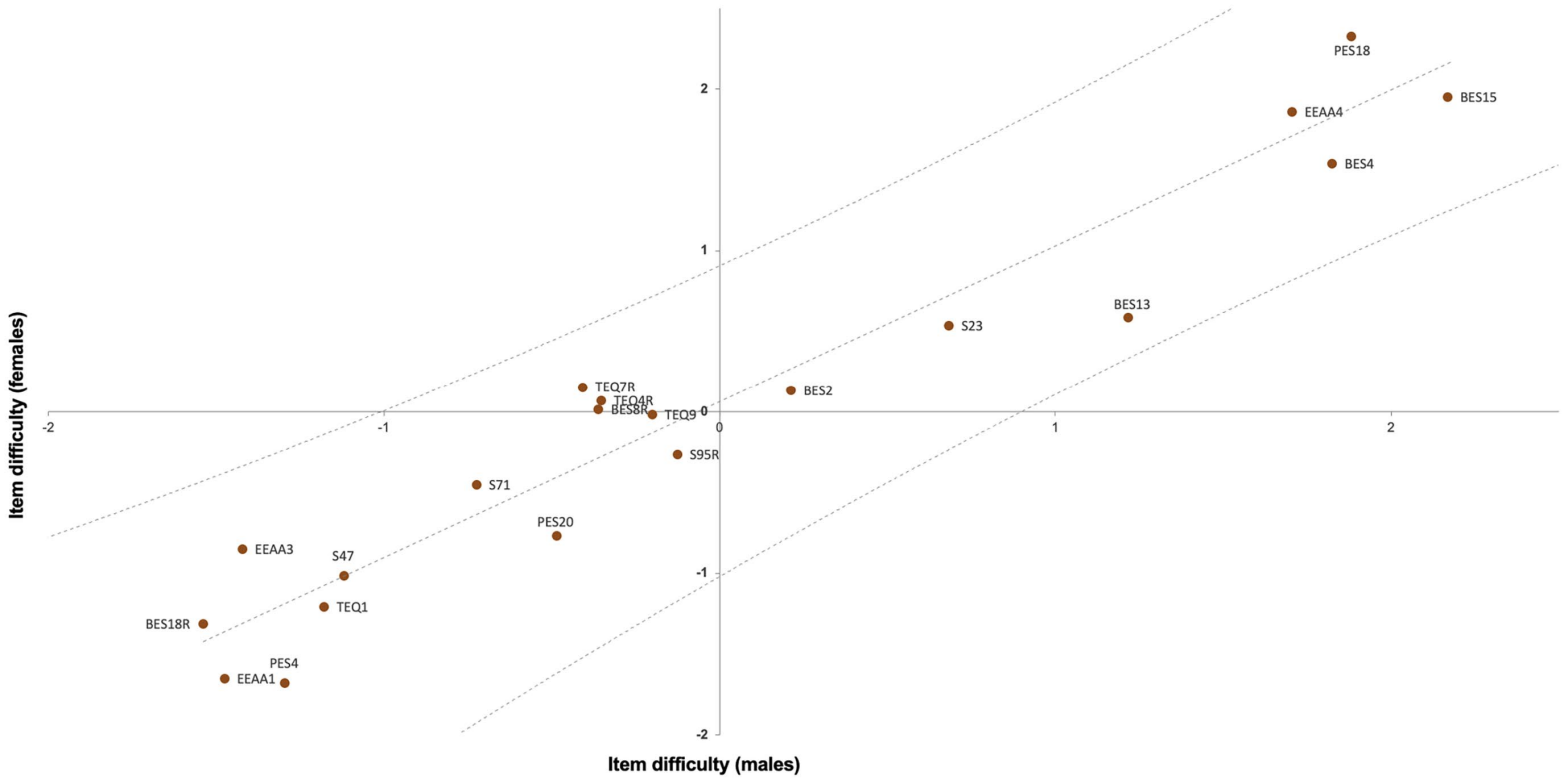
Graphical representation of the results of gender-related differential item functioning in the emotional empathy scale. The trendline demonstrates the relationship between item difficulties for females and males, along with the 95% confidence intervals.

Notably, in the emotional empathy scale, there were no items with differential functioning for males and females. All items were within the 95% confidence interval of the model linking the item difficulties for females and males (Figure 1).

In Materials and Methods section, we presented several validity criteria for the scales used in the present study, as obtained in prior Chilean research. Here, we present validity criteria for the scales as obtained in the present study based on the relationship between variables under study and sociodemographic variables (Supplementary Table S1). The information is arranged in the following order: (1) emotional empathy, (2) soil conservation behaviour, (3) altruistic behaviour.

1) The following validity criteria were obtained in this study for the emotional empathy scale:

a) Female respondents exhibited a greater emotional empathy (0.45 ± 1.2) than their male counterparts (−0.42 ± 1.3) (*p* < 0.001, *d* = 0.69, here and below “*d*” refers to Cohen’s (1988) *d* value), which is consistent with prior research on gender differences in empathy (Christov-Moore et al., 2014; Jolliffe and Farrington, 2006; Raymond et al., 2025).
b) Correlation between farmers’ emotional empathy and their altruistic behaviour was positive (*r* = 0.49, *p* < 0.001), consistent with the aforementioned empathy-altruism hypothesis (Batson et al., 1991; Batson et al., 2015).

These findings highlight the scale’s utility in assessing emotional empathy across individuals. Therefore, the measure used in this study for emotional empathy is a valid measure, which can be employed in future research.

2) The following validity criteria were obtained for the soil conservation behaviour scale:

a) Observed behaviour correlation: Positive and relatively large correlations (Supplementary Table S6) were found between farmers’ self-reported behaviours and those observed by surveyors during farm visits, indicating the scale accurately captures actual practices.
b) Formal education: Farmers with formal education in soil management (obtained via training courses or at professional institutes or universities) scored significantly higher on soil conservation behaviour (0.22 ± 1.3) than those without such training (−0.20 ± 1.4) (*p* = 0.019, *d* = 0.32).

3) The following validity criteria were obtained for the altruistic behaviour scale:

a) Membership in humanitarian organizations: Humanitarian organizations members scored higher on altruism (0.89 ± 1.2) compared to non-members (−0.16 ± 1.5) (*p* < 0.001, *d* = 0.47).
b) Gender differences: Female respondents exhibited greater altruism (0.12 ± 1.4) than their male counterparts (−0.22 ± 1.6) (*p* < 0.001, *d* = 0.24), which is consistent with prior research on gender differences in altruism (Brañas-Garza et al., 2018; Rand et al., 2016).
c) Indigenous background: Non-indigenous farmers exhibited a greater altruism (0.17 ± 1.5) than their indigenous counterparts (−0.30 ± 1.4) (*p* < 0.001, *d* = 0.32), which is consistent with prior research on racial differences in altruism (Hsieh and Lin, 2024; Lynn, 2020).

### Relationships between variables under study

A positive correlation emerged between emotional empathy and altruistic behaviour (*r* = 0.49, *p* < 0.001, Table 1). However, a negative correlation emerged between emotional empathy and engagement in soil conservation (*r* = −0.24, *p* < 0.001). Furthermore, the link between soil conservation behaviour and altruistic behaviour was not statistically significant (*r* = −0.06, *p* = 0.36).

When controlling for sociodemographic characteristics (age, gender, educational level, income level, indigenous status, and membership in humanitarian and environmental organizations), the relationships between variables under study remained virtually unchanged (Supplementary Table S5), suggesting that these relationships are not biased by individual differences in sociodemographic characteristics.

Notably, when controlling for altruism, the relationship between emotional empathy and soil conservation actions also remained unchanged (*r* = −0.24, *p* < 0.001), suggesting that this relationship is not biased by individual differences in altruism.

It is important to mention the meta-analysis conducted by Gignac and Szodorai (2016) on correlations across the field of psychology. This meta-analysis reported that the 25th, 50th, and 75th percentiles corresponded to correlations of 0.11, 0.19, and 0.29, respectively. Likewise, only 3% of correlations in the psychological literature were found to be larger than *r* = 0.50. Consequently, the relationships observed in this study are quite typical for the field.

**Figure 2:**
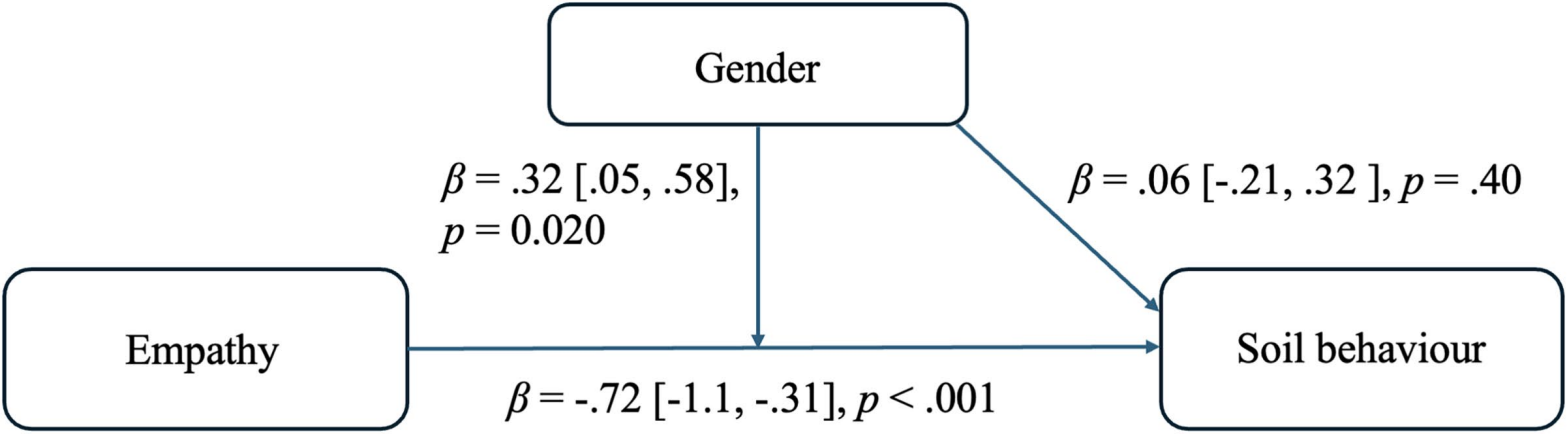
Statistical diagram showing the moderation model, whereby the effect of emotional empathy on soil conservation behaviour is moderated by the respondents’ gender (*p* = 0.020). Notably, in the database males were coded as 1 and females as 2.

Finally, there was a moderating effect of participant’s gender on the relationship between emotional empathy and soil conservation behaviour. Specifically, empathy was a significant predictor of soil conservation behaviour (*β* = −0.72, *p* < 0.001), while gender was not a significant predictor of soil conservation behaviour (*β* = 0.06, *p* = 0.40). The interaction term emotional empathy × gender was a significant predictor of soil conservation behaviour (*β* = 0.32, *p* = 0.020) (Figure 2).

**Figure 3:**
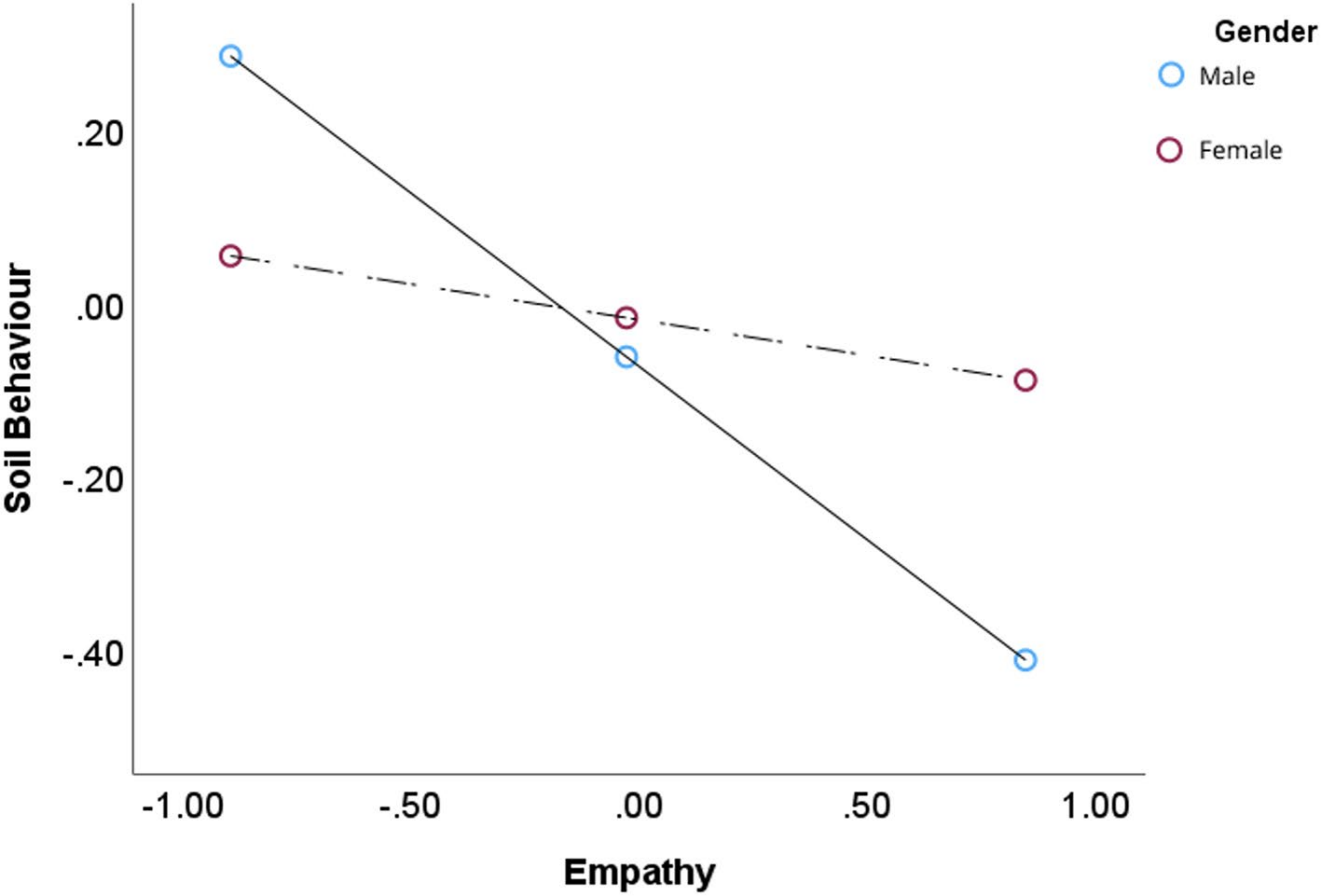
The simple slopes illustrating the relationship between emotional empathy and soil conservation behaviour at low (−1 SD), medium, and high (+1 SD) values of emotional empathy, for males and females.

Notably, Pearson correlation between emotional empathy and soil conservation was negative and significant in males (*r* = −0.37, *p* < 0.001), but not significant in females (*r* = −0.08, *p* = 0.40). Likewise, the simple slopes (Figure 3), performed in SPSS 29, illustrated the relationship between emotional empathy and soil conservation behaviour for males (*β* = −0.39 [−0.58, −0.22], *p* < 0.001) and females (*β* = −0.08 [−0.28, 0.12], *p* = 0.41).

When controlling for sociodemographic characteristics (age, educational level, income level, indigenous status, and membership in humanitarian and environmental organizations), the moderation effect by gender and simple slopes remained virtually unchanged (Supplementary Figures S1, S2), suggesting that moderation effect by gender is not biased by individual differences in sociodemographic characteristics.

## Discussion

### Interpretation of study results

The findings of the present study provide support for the empathy-altruism hypothesis (Batson et al., 1991). Specifically, this study suggests that empathy is a pre-requisite for altruistic actions. Furthermore, the positive correlation between emotional empathy and altruism observed in this study is consistent with prior Chilean research demonstrating a positive correlation between emotional empathy and pro-environmental behaviour in a sample of general Chilean population (Neaman et al., 2022).

However, the findings of the present study do not support the empathy-sustainability hypothesis proposed by Brown et al. (2019). Negative correlation between emotional empathy and soil conservation behaviour may be due to the fact that one’s behavioural options are inherently limited (Otto et al., 2021). Thus, feeling empathic towards other people may limit opportunities to feel empathic towards soil, having negative impacts on soil conservation behaviour.

Another possible explanation for the negative relationship between emotional empathy and soil conservation behaviour is that participants may have conceptualised soil primarily in relation to human outcomes, such as crop productivity, food security, or community well-being, rather than as a natural entity in itself. In those who are more empathetic, environmental degradation may, therefore, evoke distress because of its implications for human suffering and livelihood insecurity. However, such emotional reactions may not necessarily translate into soil-protective practices, particularly where economic pressures or agricultural demands may limit behavioural action. This is akin to research demonstrating that the distressing emotion of climate change anxiety does not predict pro-environmental behaviour engagement (Clayton and Karazsia, 2020). This interpretation further highlights the importance of distinguishing between empathy directed toward humans and empathy directed toward nature.

Notably, the negative relationship between emotional empathy and soil conservation was more pronounced in male compared to female farmers. Possible mechanisms may include gender-related roles in agricultural labour, farm decision-making responsibilities, economic pressures, and access to soil management knowledge (Cheong et al., 2025; Clark et al., 2023; Kassoh et al., 2026; Piedrahita et al., 2025; Thomas et al., 2025). However, the exact underlying mechanism is not known, warranting future studies. It is worth emphasizing that gender differences observed in this study are not due to differential item functioning of the empathy scale, assuring equity and fairness of assessment (Wyse and Mapuranga, 2009).

Furthermore, the findings of the present study contradict the observed positive correlation between emotional empathy and pro-environmental behaviour in Latin American and Russian samples in the study of Neaman et al. (2022). It is not possible to pinpoint the exact mechanisms behind this difference because the study of Neaman et al. (2022) and the present one differs in (1) scale used to assess emotional empathy, (2) response variable (general pro-environmental behaviour versus specific soil conservation behaviour), (3) population (general population versus specific population of farmers), and (4) countries involved (three Latin American countries and Russia versus Chile). Future studies are warranted to examine relationship between emotional empathy and pro-environmental behaviour in a sample of general Chilean population, using the same scale of emotional empathy as in the present study.

Nevertheless, from a practical perspective, the present study suggests that fostering emotional empathy with humans may not be helpful in solving current soil degradation issues.

### Limitations and future research needs

While this study advances the understanding of empathy-sustainability relationship, key limitations require consideration. The convenience sampling approach may impact the generalizability of findings by sampling concentrated regions, at one time point, in one country. Future research would benefit from replicating these findings in broader samples and contexts.

Also, this study is somewhat limited because it focused only on the emotional aspects of empathy. Existing empathy scales (Brett et al., 2023; Jolliffe and Farrington, 2006) include items reflecting cognitive empathy. Future studies are warranted to examine different (cognitive versus emotional) aspects of empathy, allowing to detect possible differential impacts of emotional empathy and cognitive empathy on pro-environmental behaviours.

Likewise, future analysis of empathy-sustainability hypothesis would benefit from including the following three measures: “empathy with nature,” “cognitive empathy with humans,” and “emotional empathy with humans.”

Furthermore, we acknowledge that our Rasch-type scale of emotional empathy can be further improved by including some more items, for instance from the Questionnaire of Cognitive and Affective Empathy (Reniers et al., 2011). Including some additional very easy and very difficult items would be the most beneficial since it will improve the sensitivity of the scale to discern between individuals with contrasting levels of emotional empathy.

Moreover, this study focused only on empathy with humans. Future research should more clearly differentiate the object of empathy under investigation, including empathy toward humans, and nature. Specifically, future research should examine the relationship between soil conservation behaviour and empathy with nature, which might have a closer link to nature conservation compared to empathy with humans.

It may also be valuable to examine how farmers cognitively represent “soil” and environmental degradation, and whether these representations may impact how empathy relates to conservation behaviour. Likewise, future studies may also assess gender-related roles in agricultural labour, farm decision-making responsibilities, economic pressures, and access to soil management knowledge.

Furthermore, the present study is limited to one type of pro-environmental behaviour, namely soil conservation behaviour, and a specific population of farmers. Future studies may consider other types of pro-environmental behaviour and other populations.

Finally, this study is solely based on a Chilean sample, yet, several studies in environmental psychology have documented country-level differences (Aydin et al., 2022; Schultz et al., 2004; Schultz et al., 2005), thus warranting future studies in other Spanish-speaking countries.

## Conclusion

This study delivers the first report on the relationship between emotional empathy and soil conservation behaviour. Likewise, it is the first report on the effect of emotional empathy on both altruistic and soil conservation actions in a population of farmers. Furthermore, it is the first report on the moderating effect of gender on the relationship between farmers’ emotional empathy and their soil conservation practices.

Although the findings of the present study support the empathy-altruism hypothesis (Batson et al., 1991), the present study suggests that fostering emotional empathy with humans may not be helpful in solving current soil degradation issues, since a negative correlation emerged between emotional empathy and soil conservation behaviour.

## Data Availability

The raw data supporting the conclusions of this article will be made available by the authors, without undue reservation.
