# Supplementary material for: Helping people, neglecting soil: contrasting effects of emotional empathy with humans on altruistic behaviour and soil conservation in a population of Chilean farmers
Source: Front Psychol. 2026 Jul 9;17:1843293. doi: 10.3389/fpsyg.2026.1843293 (PMC13391298; doi:10.3389/fpsyg.2026.1843293)
Supplement: Supplementary file 1 [file Supplementary_file_1.docx]

**Supplementary Material**

**The validity criteria for the soil conservation behaviour scale obtained in prior Chilean studies**

a) **Observed behaviour correlation:** Farmers’ self-reported behaviours demonstrated strong positive correlations with real situations as documented by the surveyors at the farms (Burnham et al., 2023; Neaman et al., 2025).

b) **Formal education:** Farmers who reported receiving formal soil management education (university, professional institute, high school, or training courses) displayed statistically higher levels of soil conservation behaviour compared to those without formal education (Burnham et al., 2023; Neaman et al., 2025). The research of Neaman et al. (2024) revealed that Chilean farmers with formal agricultural training had much better soil science understanding than those without, showing specific, structured education effectively builds deep knowledge, unlike broader experience-based environmental awareness (Otto & Kaiser, 2014), emphasizing the need for tailored learning for specialized skills. This underscores the unique focus of soil conservation behaviour research, emphasizing the need for a deeper understanding of soil-specific concepts. This aligns with the “knowledge-deficit theory” (Schultz, 2002), which suggests that a lack of knowledge can contribute to inaction.

c) Ecological farm management: In the study by Burnham et al. (2023), farmers who reported utilizing ecological management practices on their farm exhibited superior soil conservation behaviour compared to their non-ecologically minded counterparts.

**The validity criteria for the altruism scale obtained in prior Chilean studies**

a) Convergent validity: The honesty-humility scale from the HEXACO personality inventory (Lee & Ashton, 2018) was validated in Spanish by Roncero et al. (2013) and Romero et al. (2015). The honesty-humility trait reflects cooperation and fairness, suggesting an underlying prosociality (Ashton & Lee, 2007; Pfattheicher & Böhm, 2018). A positive relationship between altruism and honesty-humility has been consistently demonstrated in Chilean studies (Neaman et al., 2022; Otto et al., 2021).

b) Membership in humanitarian organizations: In previous Chilean research (Otto et al., 2021), members of humanitarian organizations (i.e., organizations aiming to providing social help for other people) scored higher on altruism compared to non-members (ANOVA, *p* < .001).

**
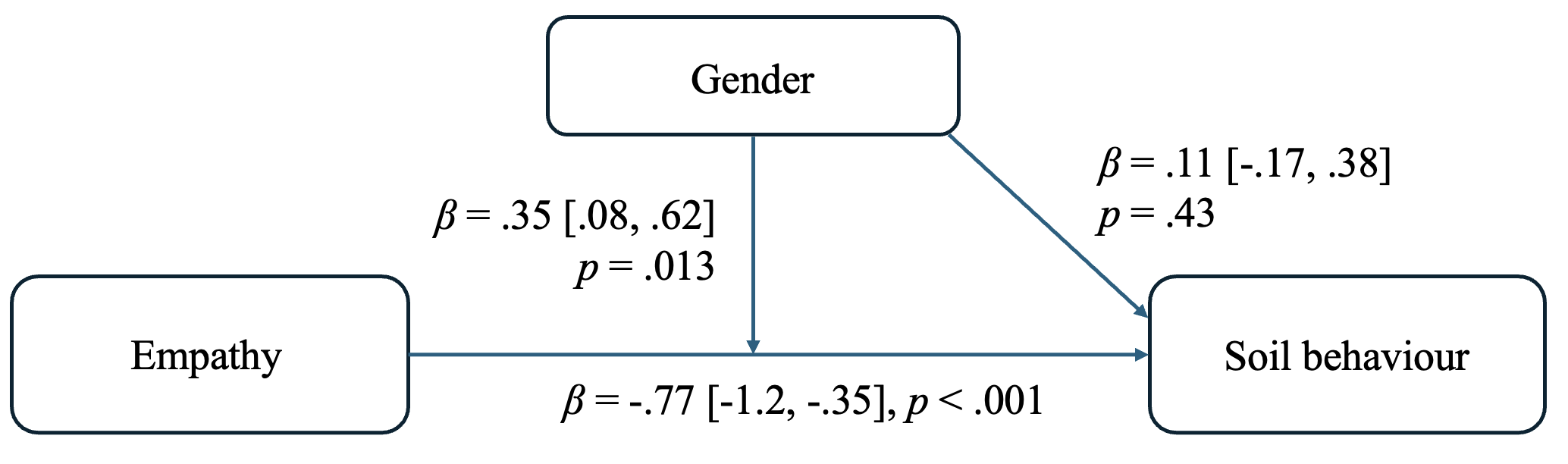
**

**Supplementary Figure 1.** Statistical diagram showing the moderation model, whereby the effect of emotional empathy on soil conservation behavior is moderated by the respondents’ gender (*p* = 0.013). Notably, in the database males were coded as 1 and females as 2.


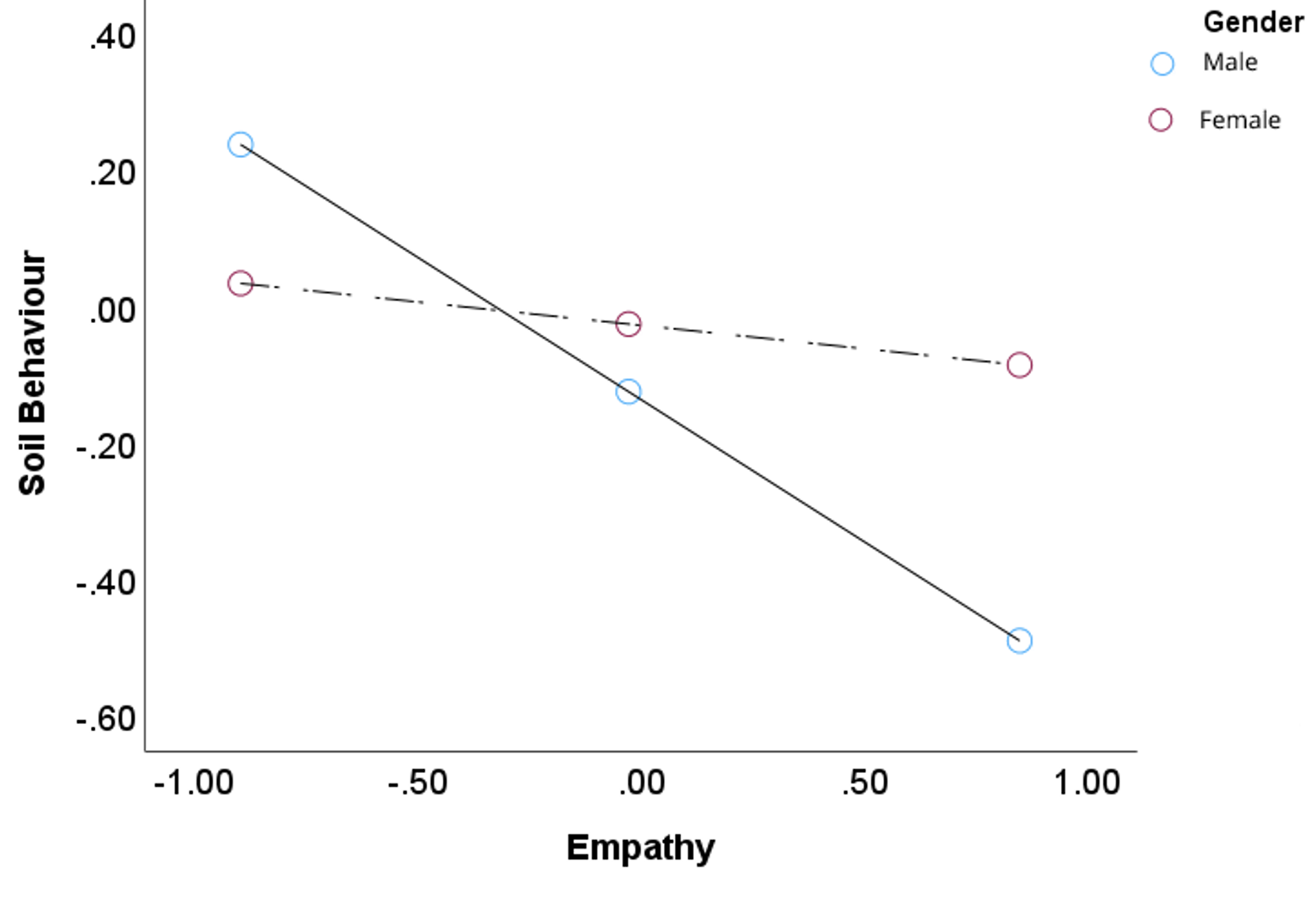


**Supplementary Figure 2.** The simple slopes illustrating the relationship between emotional empathy and soil conservation behavior at low (-1 SD), medium, and high (+1 SD) values of emotional empathy, for males (*β =* -.42 [-.60, -.23], *p* < .001) and females (*β* = -.07 [-.28, .14], *p* = .51).

**Supplementary Table 1.** Sociodemographic characteristics of participants. The age range was 18–82 years old (*M* ± *SD* of 49 ± 15). The education level was 3.8 ± 1.6 (range 1–7), whereas the income level was 2.1 ± 0.59 (range 1–3).

| Variable | Category | % |
| --- | --- | --- |
| Gender, % | Male | 52 |
|  | Female | 47 |
|  | Not reported | 1 |
| Occupation, % | Independent farmer | 73 |
|  | Employee in an agricultural company | 7 |
|  | Owner of an agricultural company | 10 |
|  | Other | 9 |
|  | Not reported | 1 |
| Formal education on soil management, % | Yes^1^ | 42 |
|  | No | 55 |
|  | Not reported | 3 |
| Indigenous ethnicity, % | No | 49 |
|  | Yes, Mapuche | 9 |
|  | Yes, Aymara | 35 |
|  | Yes, Quechua | 2 |
|  | Yes, another ethnical group | 1 |
|  | Not reported | 4 |
| Member of a humanitarian | No | 88 |
| organization, % | Yes | 11 |
|  | Not reported | 1 |
| Member of an environmental | No | 93 |
| organization, % | Yes | 5 |
|  | Not reported | 2 |

^1^ University, professional institute, training course(s) or similar

**Supplementary Table 2a.** The empathy scale used in this study. Items in *italics* were negatively formulated and reverse coded before analysis. These items should be read as ‘I refrain from...’.

| Nº | Item | MS | δ |
| --- | --- | --- | --- |
| PES14 | When I see or hear someone who is disgusted, it makes me feel disgusted too. | 1.04 | 1.51 |
| PES18 | When I see or hear someone who is embarrassed, it makes me feel embarrassed too. | 0.92 | 1.27 |
| BES15 | I tend to feel scared when I am with friends who are afraid. | 0.95 | 1.18 |
| EEAA4 | Depressed people bring me down. | 1.06 | 0.91 |
| BES4 | I get frightened when I watch characters in a good scary movie. | 1.00 | 0.84 |
| BES13 | Seeing a person who has been angered has no effect on my feelings. | 0.97 | 0.15 |
| S23 | I feel like crying when I see other people crying. | 0.89 | -0.15 |
| BES2 | After being with a friend who is sad about something, I usually feel sad. | 0.87 | -0.55 |
| TEQ9 | I find that I am “in tune” with other people’s moods. | 0.85 | -0.83 |
| *TEQ7R* | *When a friend starts to talk about his/her problems, I try to steer the conversation towards something else.* | 1.23 | -0.85 |
| *TEQ4R* | *I remain unaffected when someone close to me is happy.* | 0.97 | -0.85 |
| *BES8R* | *Other people’s feelings don’t bother me at all.* | 1.03 | -0.88 |
| *S95R* | *I remain unemotional even in situations where most people get very sentimental.* | 0.93 | -0.89 |
| PES20 | When I see or hear someone who is proud, it makes me feel proud too. | 0.98 | -1.27 |
| S71 | I feel strong emotions when someone close to me is going away for a long time. | 0.99 | -1.29 |
| S47 | When someone I know well is unhappy, I can almost feel that person's pain myself. | 0.90 | -1.72 |
| EEAA3 | Laugh when others laugh. | 1.02 | -1.79 |
| TEQ1 | When someone else is feeling excited, I tend to get excited too. | 0.97 | -1.82 |
| *BES18R* | *My friend’s unhappiness doesn’t make me feel anything.* | 1.00 | -2.03 |
| PES4 | When I see or hear someone who is happy, it makes me feel happy too. | 0.85 | -2.04 |
| EEAA1 | Suffering of others disturbs me. | 0.90 | -2.14 |

*Note:* Here and below, δ = item difficulty, MS = infit mean square,

BES = Basic Empathy Scale (Jolliffe & Farrington, 2006),

EEAA = measure of Emotional Empathy for Adolescents and Adults (Caruso & Mayer, 1998),

PES = the Perth Empathy Scale (Brett et al., 2023),

TEQ = Toronto Empathy Questionnaire (Spreng et al., 2009);

S = the sentimentality scale of the HEXACO personality inventory (Lee & Ashton, 2018).

**Supplementary Table 2b.** The empathy scale used in the study. Here and below, the original Spanish is followed by the English translation.

| Nº | Original Spanish item | English translation |
| --- | --- | --- |
| PES4 | Cuando veo o escucho a alguien feliz, me siento feliz también. | When I see or hear someone who is happy, it makes me feel happy too. |
| PES14 | Cuando veo o escucho a alguien que está disgustado, siento disgusto también. | When I see or hear someone who is disgusted, it makes me feel disgusted too. |
| PES18 | Cuando veo o escucho a alguien que está avergonzado/a, también siento vergüenza. | When I see or hear someone who is embarrassed, it makes me feel embarrassed too. |
| PES20 | Cuando veo o escucho a alguien que está orgulloso, también me siento orgulloso/a. | When I see or hear someone who is proud, it makes me feel proud too. |
| BES2 | Después de estar con un/a amigo/a que está triste por algo, normalmente me siento triste. | After being with a friend who is sad about something, I usually feel sad. |
| BES4 | Me dan miedo algunos personajes de las películas de terror. | I get frightened when I watch characters in a good scary movie. |
| *BES8R* | *Los sentimientos de otras personas me son indiferentes.* | *Other people’s feelings don’t bother me at all.* |
| BES13 | Ver a una persona muy enfadada me afecta emocionalmente. | Seeing a person who has been angered has no effect on my feelings. |
| BES15 | Suelo sentir miedo cuando mis amigos tienen miedo. | I tend to feel scared when I am with friends who are afraid. |
| *BES18R* | *Las desgracias de mis amigos/as me son indiferentes.* | *My friend’s unhappiness doesn’t make me feel anything.* |
| S23 | Siento ganas de llorar cuando veo llorar a otras personas. | I feel like crying when I see other people crying. |
| S47 | Cuando alguien muy cercano a mí es infeliz, puedo sentir el dolor de esa persona. | When someone I know well is unhappy, I can almost feel that person's pain myself. |
| S71 | Siento emociones fuertes, cuando alguien cercano a mí se va a alejar por un largo tiempo. | I feel strong emotions when someone close to me is going away for a long time. |
| *S95R* | *No me emociono incluso en situaciones donde la mayoría de las personas se ponen muy sentimentales.* | *I remain unemotional even in situations where most people get very sentimental.* |
| TEQ1 | Cuando otras personas demuestran entusiasmo, tiendo a sentirme entusiasmado/a también. | When someone else is feeling excited, I tend to get excited too. |
| *TEQ4R* | *Cuando alguien cercano a mí está contento, mi estado no cambia.* | *I remain unaffected when someone close to me is happy.* |
| *TEQ7R* | *Cuando un/a amigo/a empieza a contarme sus problemas, trato de cambiar de tema.* | *When a friend starts to talk about his/her problems, I try to steer the conversation towards something else.* |
| TEQ9 | En general, “sintonizo” con los estados de ánimo de los demás. | I find that I am “in tune” with other people’s moods. |
| EEAA1 | Me duele ver a una persona sufrir. | Suffering of others disturbs me. |
| EEAA3 | Me río cuando otras personas se ríen. | Laugh when others laugh. |
| EEAA4 | La gente deprimida me deprime. | Depressed people bring me down. |

**Supplementary Table 3a.** The soil conservation behavior scale used in this study.

| Nº | Item | MS | δ |
| --- | --- | --- | --- |
| SB16 | For phosphate fertilizers that I intend to apply to the soil, I conduct heavy metal (cadmium, lead, etc.) analysis. | 0.92 | 2.60 |
| SB19 | I inoculate the soil with symbiotic fungi (Mycorrhizae). | 0.90 | 1.53 |
| SB04 | I perform chemical analysis of organic matter to be applied to the soil. | 0.90 | 1.53 |
| SB20 | In calculating the dosage of fertilizers to be applied to the soil, I consider the nutrient contribution from irrigation water. | 0.87 | 1.12 |
| SB22 | On the sloped parts of my property, I practice terrace farming. | 0.98 | 1.10 |
| SB07 | I incorporate worms into the soil. | 0.95 | 0.95 |
| SB11 | I apply *Rhizobium* bacteria and/or other beneficial microorganisms to the soil. | 0.99 | 0.95 |
| SB18 | I calculate the dosage of fertilizers to be applied based on the soil analysis results. | 0.89 | 0.84 |
| SB13 | I create compost with agricultural waste. | 0.97 | 0.02 |
| SB30 | I use biological pesticides (herbicides, fungicides, or nematicides) on the farm. | 1.05 | 0.02 |
| SB24 | I perform solarization, which is the soil disinfection method using heat generated by solar energy. | 1.20 | 0.01 |
| SB21 | I maintain live vegetative cover crops on the property. | 0.99 | -0.28 |
| SB25 | I apply fermented organic manure to the soil. | 1.00 | -0.57 |
| SB28 | I leave some plots fallow, meaning I leave some land unused for at least a year. | 1.04 | -0.57 |
| SB02 | I apply compost to the soil. | 0.95 | -0.61 |
| SB35 | I restore eroded soils on my property. | 0.93 | -1.20 |
| SB39 | I practice crop rotation on my farm. | 0.94 | -1.26 |

**Supplementary Table 3b.** The soil conservation behavior scale used in study.

| Nº | Original Spanish item | English translation |
| --- | --- | --- |
| SB2 | Aplico compost al suelo. | I apply compost to the soil. |
| SB4 | Realizo análisis químico de la materia orgánica que será aplicada al suelo. | I perform chemical analysis of organic matter to be applied to the soil. |
| SB7 | Incorporo lombrices al suelo. | I incorporate worms into the soil. |
| SB11 | Aplico al suelo bacterias *Rhizobium* y/u otros microorganismos benéficos. | I apply *Rhizobium* bacteria and/or other beneficial microorganisms to the soil. |
| SB13 | Realizo compost con los desechos agrícolas de mi predio. | I create compost with agricultural waste. |
| SB30 | En el predio, aplico pesticidas (o herbicidas o fungicidas o nematicidas) de origen biológico. | I use biological pesticides (herbicides, fungicides, or nematicides) on the farm. |
| SB35 | Recupero suelos erosionados en mi predio. | I restore eroded soils on my property. |
| SB39 | En mi predio, practico la rotación de cultivos. | I practice crop rotation on my farm. |
| SB16 | En los fertilizantes fosfatados que voy a aplicar al suelo, realizo análisis de metales pesados (cadmio, plomo, etc.). | For phosphate fertilizers that I intend to apply to the soil, I conduct heavy metal (cadmium, lead, etc.) analysis. |
| SB18 | Calculo la dosis de fertilizantes que serán aplicados, basándome en los resultados de análisis del suelo. | I calculate the dosage of fertilizers to be applied based on the soil analysis results. |
| SB19 | Inoculo el suelo con hongos simbióticos (micorrizas). | I inoculate the soil with symbiotic fungi (Mycorrhizae). |
| SB20 | En el cálculo de la dosis de fertilizantes que serán aplicados al suelo, considero el aporte de nutrientes del agua de riego. | In calculating the dosage of fertilizers to be applied to the soil, I consider the nutrient contribution from irrigation water. |
| SB21 | Mantengo cultivos de cobertura vegetal viva en el predio. | I maintain live vegetative cover crops on the property. |
| SB22 | En partes inclinadas de mi predio, realizo cultivo en terrazas / andenes. | On the sloped parts of my property, I practice terrace farming. |
| SB24 | Realizo la solarización, es decir, la desinfección del suelo por medio del calor que genera la energía solar. | I perform solarization, which is the soil disinfection method using heat generated by solar energy. |
| SB25 | Aplico abonos orgánicos fermentados al suelo. | I apply fermented organic manure to the soil. |
| SB28 | Dejo algunos terrenos en barbecho, es decir, dejo algunos terrenos sin uso por lo menos un año. | I leave some plots fallow, meaning I leave some land unused for at least a year. |

**Supplementary Table 4a.** The altruism scale used scale used in this study.

| Nº | Item | MS | δ |
| --- | --- | --- | --- |
| A08 | I donate blood. | 0.96 | 3.12 |
| A12 | I give a stranger a lift in my car. | 1.01 | 2.48 |
| A07 | I do volunteer work for a charity. | 0.99 | 2.30 |
| A22 | I put myself at risk to help someone I don’t know. | 0.99 | 2.18 |
| A03 | I change money for a stranger. | 1.00 | 2.17 |
| A18 | I comfort a stranger who was crying. | 1.02 | 1.65 |
| A25 | I spend time with strangers who seemed to be alone at an event. | 0.98 | 1.59 |
| A05 | I give money to a stranger in need. | 0.88 | 1.42 |
| A24 | When I see a stranger being mocked on the street, I defend them. | 1.00 | 1.37 |
| A21 | I am kind to strangers who seem unfriendly or rude at first. | 1.07 | 1.32 |
| A06 | I donate goods or clothes to a charity. | 0.91 | 0.93 |
| A04 | I give money to a charity or fundraising campaign to help somebody. | 0.96 | 0.63 |
| A01 | I help push a stranger’s broken car. | 1.03 | 0.50 |
| A09 | I help carry a stranger’s belongings (bags, parcels, etc.). | 0.88 | 0.43 |
| A10 | I delay an elevator and hold the door open for a stranger. | 0.93 | 0.04 |
| A11 | I allow someone to go ahead of me in a lineup (driving a car, in the supermarket). | 0.95 | 0.01 |
| A14 | I buy a “Teletón^1^” product. | 1.12 | -0.11 |
| A17 | I offer my seat on a bus or train to a stranger who is standing. | 0.99 | -0.91 |
| A02 | I give directions to a stranger. | 0.90 | -1.09 |
| A16 | I offer to help elderly strangers cross the street. | 0.83 | -1.24 |
| A19 | I help an unknown person who fell on the street. | 0.77 | -1.39 |
| A13 | When I receive extra change, I give it back to the cashier | 1.06 | -1.47 |
| A26 | If a stranger drops something, I pick it up and give it back to them. | 0.81 | -2.06 |

^1^ In Chile, *Teletón* (a massive telethon) is a nationwide, annual charitable event.

**Supplementary Table 4b.** The altruism scale used scale used in this study

| Nº | Original Spanish item | English translation |
| --- | --- | --- |
| A1 | Ayudo a empujar un auto en pana conducido por una persona desconocida. | I have helped push a stranger’s broken car. |
| A2 | Explico a una persona desconocida cómo llegar a un lugar. | I have given directions to a stranger. |
| A3 | Cambio dinero a una persona desconocida. | I have made change for a stranger. |
| A4 | Dono dinero a una organización de beneficencia o a una campaña de colecta de dinero para ayudar a otros. | I have given money to a charity or fundraising campaign to help somebody. |
| A5 | Entrego dinero a una persona desconocida que lo necesita. | I have given money to a stranger who needed it (or asked me for it). |
| A6 | Dono alimentos o ropa como caridad. | I have donated goods or clothes to a charity. |
| A7 | Hago trabajos voluntarios como caridad. | I have done volunteer work for a charity. |
| A8 | Dono sangre. | I have donated blood. |
| A9 | Ayudo a cargar cosas (maletas, bolsos, etc.) a una persona desconocida. | I have helped carry a stranger’s belongings (bags, parcels, etc.). |
| A10 | Detengo un ascensor y mantengo sus puertas abiertas para una persona desconocida. | I have delayed an elevator and held the door open for a stranger. |
| A11 | Le doy la preferencia a una persona desconocida (en una fila, manejando auto, etc.). | I have allowed someone to go ahead of me in a lineup (driving a car, in the supermarket). |
| A12 | Llevo en mi auto a una persona desconocida. | I have given a stranger a lift in my car. |
| A13 | Al recibir vuelto demás en una caja, le devuelvo el dinero extra al cajero. | When I receive extra change, I give it back to the cashier |
| A14 | Compro un producto adherido a la Teletón. | I have bought a “Teletón^1^” product. |
| A16 | Ayudo a una persona desconocida (por ejemplo, anciana) a cruzar la calle. | I have offered to help a handicapped or elderly stranger across a street. |
| A17 | Cedo mi asiento a una persona desconocida, en un bus o en el metro. | I have offered my seat on a bus or train to a stranger who was standing. |
| A18 | Consuelo a una persona desconocida que está llorando. | I comfort a stranger who was crying. |
| A19 | Ayudo a una persona desconocida que se cayó en la calle. | I help an unknown person who fell on the street. |
| A21 | Soy amable con las personas desconocidas que parecen antipáticas o groseras a primera vista. | I am kind to strangers who seem unfriendly or rude at first. |
| A22 | Me pongo en una situación de riesgo para ayudar a alguien que no conozco. | I put myself at risk to help someone I don’t know. |
| A24 | Cuando veo a una persona desconocida en la calle siendo objeto de burlas, defiendo a esa persona. | When I see a stranger being mocked on the street, I defend them. |
| A25 | Paso tiempo con personas desconocidas que parecían estar solas en un evento. | I spend time with strangers who seemed to be alone at an event. |
| A26 | Si a una persona desconocida se le cae algo, lo recojo y se lo devuelvo. | If a stranger drops something, I pick it up and give it back to them. |

^1^ In Chile, *Teletón* (a massive telethon) is a nationwide, annual charitable event.

**Supplementary Table 5.** Partial correlations (controlled for socio-demographical variables) between the variables under study.

| Variable | 1 | 2 | 3 |
| --- | --- | --- | --- |
| 1. Empathy | – |  |  |
|  |  |  |  |
| 2. Soil conservation behaviour | -.24*** | – |  |
|  | [-.12, -.35] |  |  |
| 3. Altruistic behaviour | .48*** | n.s. | – |
|  | [.38, .56] |  |  |

Values in square brackets indicate the 95% confidence interval for each correlation

n.s. = not statistically significant, ^***^ *p* < .001

**Supplementary Table 6.** Pearson correlation coefficients (*r*) between self-reported behaviour and observed (actual) behaviour in farmers, as used in the study.

| Nº | Observed (real) behaviour | Nº | Self-reported behaviour | *r* | *p* |
| --- | --- | --- | --- | --- | --- |
| V1 | The use of cover crops on the property is observed. | SB21 | I maintain live vegetative cover crops on the property. | .20 | .02 |
| V3 | The use of fallow land is observed. | SB28 | I leave some plots fallow, meaning I leave some land unused for at least a year. | .55 | < .001 |
| V7 | Areas with vegetal materials undergoing composting are observed. | SB13 | I create compost with agricultural waste. | .64 | < .001 |
| V8 | Soil disinfection through solarization is evident. | SB24 | I perform solarization, which is the soil disinfection method using heat generated by solar energy. | .59 | < .001 |
| V10 | The farmer uses biological pesticides/herbicides/fungicides/nematicides. | SB30 | I use biological pesticides (herbicides, fungicides, or nematicides) on the farm. | .47 | < .001 |
| V12 | It is evident that the farmer produces fermented organic fertilizers and/or liquid humus. | SB25 | I apply fermented organic manure to the soil. | .45 | < .001 |
| V13 | On sloping parts of the property, terrace cultivation or raised beds following contour lines are evident. | SB22 | On the sloped parts of my property, I practice terrace farming. | .70 | < .001 |

**References**

Ashton, M. C., & Lee, K. (2007). Empirical, Theoretical, and Practical Advantages of the HEXACO Model of Personality Structure. *Personality and Social Psychology Review*, *11*, 150–166. <https://doi.org/10.1177/1088868306294907>

Brett, J. D., Becerra, R., Maybery, M. T., & Preece, D. A. (2023). The psychometric assessment of empathy: Development and validation of the Perth Empathy Scale. *Assessment*, *30*(4), 1140–1156. <https://doi.org/10.1177/10731911221086987>

Burnham, E., Zabel, S., Navarro-Villarroel, C., Ermakov, D. S., Castro, M., Neaman, A., & Otto, S. (2023). Enhancing farmers’ soil conservation behavior: beyond soil science knowledge. *Geoderma*, *437*, 116583. <https://doi.org/10.1016/j.geoderma.2023.116583>

Caruso, D. R., & Mayer, J. D. (1998). A Measure of Emotional Empathy for Adolescents and Adults. *Unpublished Manuscript, available at* [*https://scholars.unh.edu/cgi/viewcontent.cgi?article=1021&context=personality_lab*](https://scholars.unh.edu/cgi/viewcontent.cgi?article=1021&context=personality_lab).

Jolliffe, D., & Farrington, D. P. (2006). Development and validation of the Basic Empathy Scale. *Journal of adolescence*, *29*(4), 589–611. <https://doi.org/10.1016/j.adolescence.2005.08.010>

Lee, K., & Ashton, M. C. (2018). Psychometric Properties of the HEXACO-100. *Assessment*, *25*(5), 543–556. <https://doi.org/10.1177/1073191116659134>

Neaman, A., Baierl, T.-M., Navarro-Villarroel, C., Poblete-Ramos, F., Lizardi, N., Burnham, E., Ermakov, D. S., & Castro, M. (2025). How emotional connection empowers farmer-driven soil conservation. *Revista Brasileira de Ciencia do Solo*, *49*, e0250085. <https://doi.org/10.36783/18069657rbcs20250085>

Neaman, A., Navarro-Villarroel, C., Poblete-Ramos, F., Lizardi, N., Burnham, E., Huerta-Salinas, O., Zabel, S., D.S., E., Castro, M., & Otto, S. (2024). Reconciling the soil stewardship paradox: Knowledge without care, care without knowledge. *Geoderma Regional*, *37*, e00794. <https://doi.org/10.1016/j.geodrs.2024.e00794>

Neaman, A., Pensini, P., Zabel, S., Otto, S., Ermakov, D. S., Dovletyarova, E. A., Burnham, E., Castro, M., & Navarro-Villarroel, C. (2022). The Prosocial Driver of Ecological Behavior: The Need for an Integrated Approach to Prosocial and Environmental Education. *Sustainability*, *14*(7), 4202. <https://doi.org/10.3390/su14074202>

Otto, S., & Kaiser, F. G. (2014). Ecological behavior across the lifespan: Why environmentalism increases as people grow older. *Journal of Environmental Psychology*, *40*, 331–338. <https://doi.org/10.1016/j.jenvp.2014.08.004>

Otto, S., Pensini, P., Zabel, S., Diaz-Siefer, P., Burnham, E., Navarro-Villarroel, C., & Neaman, A. (2021). The prosocial origin of sustainable behavior: A case study in the ecological domain. *Global Environmental Change-Human and Policy Dimensions*, *69*, 102312. <https://doi.org/10.1016/j.gloenvcha.2021.102312>

Pfattheicher, S., & Böhm, R. (2018). Honesty-humility under threat: Self-uncertainty destroys trust among the nice guys. *Journal of Personality and Social Psychology*, *114*, 179 – 194. <https://doi.org/10.1037/pspp0000144>

Romero, E., Villar, P., & López-Romero, L. (2015). Assessing six factors in Spain: Validation of the HEXACO-100 in relation to the Five Factor Model and other conceptually relevant criteria. *Personality and Individual Differences*, *76*, 75–81. <https://doi.org/10.1016/j.paid.2014.11.056>

Roncero, M., Fornés, G., & Belloch, A. (2013). Hexaco: Una nueva aproximación a la evaluación de la personalidad en español. *Revista Argentina de Clínica Psicológica*, *22*(3), 205–217.

Schultz, P. W. (2002). Knowledge, information, and household recycling: Examining the knowledge-deficit model of behavior change. In T. Dietz & P. C. Stern (Eds.), *New tools for environmental protection: Education, information, and voluntary measures* (pp. 67–82). National Academy Press.

Spreng, R. N., McKinnon, M. C., Mar, R. A., & Levine, B. (2009). The Toronto Empathy Questionnaire: Scale development and initial validation of a factor-analytic solution to multiple empathy measures. *Journal of Personality Assessment*, *91*(1), 62–71. <https://doi.org/10.1080/00223890802484381>
